# Sirtuins are crucial regulators of T cell metabolism and functions

**DOI:** 10.1038/s12276-022-00739-7

**Published:** 2022-03-16

**Authors:** Imene Hamaidi, Sungjune Kim

**Affiliations:** 1 Department of Immunology, H. Lee Moffitt Cancer Center, Tampa, FL 33612 USA; 2 Department of Radiation Oncology H. Lee Moffitt Cancer Center, Tampa, FL 33612 USA

**Keywords:** Acetylation, T cells

## Abstract

It is well known that metabolism underlies T cell differentiation and functions. The pathways regulating T cell metabolism and function are interconnected, and changes in T cell metabolic activity directly impact the effector functions and fate of T cells. Thus, understanding how metabolic pathways influence immune responses and ultimately affect disease progression is paramount. Epigenetic and posttranslational modification mechanisms have been found to control immune responses and metabolic reprogramming. Sirtuins are NAD^+^-dependent histone deacetylases that play key roles during cellular responses to a variety of stresses and have recently been reported to have potential roles in immune responses. Therefore, sirtuins are of significant interest as therapeutic targets to treat immune-related diseases and enhance antitumor immunity. This review aims to illustrate the potential roles of sirtuins in different subtypes of T cells during the adaptive immune response.

## Immunity: How to give T cells a boost

Sirtuins, enzymes that regulate how cells respond to stress, regulate T cell metabolism and functions, and therefore blocking or boosting sirtuins influences immune responses. As part of the immune system, some types of T cells attack specific targets; others keep the immune response in check. Imene Hamaidi and Sungjune Kim at H. Lee Moffitt Cancer Center, Tampa, USA, have reviewed how sirtuins affect different subsets of T cells to either promote or suppress immune responses. Boosting sirtuins that increase the function of inflammation-suppressing T cells can improve outcomes for transplant recipients or help treat autoimmune diseases. Conversely, stimulating immune-activating sirtuins can help re-energize exhausted antitumor T cells. Understanding the complex web of sirtuin–T cell interactions may help in developing therapeutic strategies for improving transplant outcomes, and for treating autoimmune diseases and cancer.

## Introduction

### Overview of the T cell-mediated adaptive immune response

**Fig. 1:**
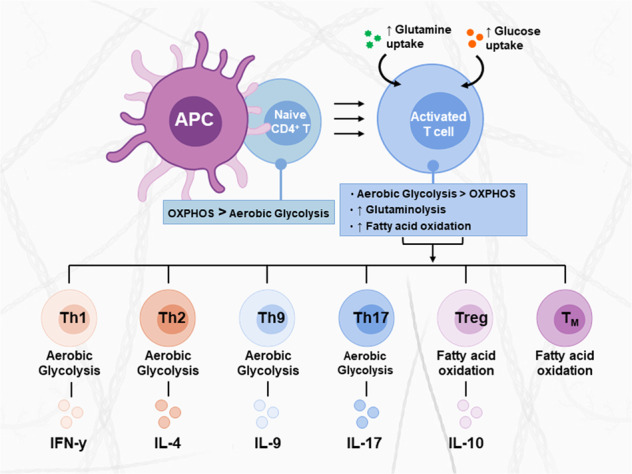
The metabolic programs of CD4^+^ T cell subsets. Distinct T cell subsets utilize specific metabolic programs to support their functions. Each functional subset is characterized by specific signaling pathways, transcription factors, metabolic programs, and effector cytokines. Naive T cells are quiescent and rely on OxPhos for their minimal energetic needs. Upon activation, activated T cells switch to aerobic glycolysis and increase glucose and glutamine uptake, which supports cell growth and proliferation. The differentiation of activated T cells into different subsets is due to several metabolic and signaling pathways. Th1, Th2, Th9, and Th17 cells primarily rely on aerobic glycolysis and glutaminolysis; in contrast, Treg and T_M_ cells upregulate fatty acid oxidation.

The adaptive immune response elicited by T cells is crucial in mounting specific immune responses against foreign pathogens. T cells develop in the thymus and, upon maturation, are classified by their expression of either CD4 or CD8 receptors. CD4^+^ and CD8^+^ T cells exist in several subsets that perform unique functions during an immune response and are characterized by specific surface receptors, cytokine secretion, and expression of lineage-defining transcription factors (Fig. 1)^1,2^. Depending on the pathogen type and the cytokines generated by antigen-presenting cells (APCs), CD4^+^ T cells can become activated and differentiate into functionally distinct T helper (Th) 1, Th2, Th17, Th9, or T regulatory (Treg) cells^3^, while CD8^+^ T cells differentiate into cytotoxic T lymphocytes (CTLs)^4^. Th1 cells, which produce interferon-gamma (IFN-γ), interleukin (IL)-2, and tumor necrosis factor-beta (TNF-β), evoke cell-mediated immunity and phagocyte-dependent inflammation and are involved in the elimination of intracellular pathogens^5^. Th2 cells, which produce IL-4, IL-5, IL-6, IL-9, IL-10, and IL-13, are known to provide immunity against extracellular parasites, including helminths, and play major roles in the induction and persistence of asthma as well as other allergic diseases^6^. Th9 cells are known to primarily produce IL-9 and facilitate the immune response against helminth parasites^7^. Th17 cells are characterized by the production of IL-17 and are involved in host protection against microbial infections that are not resolved by Th1 or Th2 immunity, including infections with a subset of extracellular bacteria and some fungi^8^. Retinoic acid receptor-related orphan nuclear receptor gamma (RORγt) is considered to be one of the master regulators of the development of Th17 cells^8^. In contrast to the proinflammatory phenotype of Th cells, Treg cells participate in the prevention of uncontrollable inflammatory responses, autoimmune diseases and allergies by suppressing T effector (T_EFF_) cells and other immune cells^9^. Treg cells are defined by the expression of the forkhead box P3 (Foxp3) transcription factor^10^. This cell subset secretes key anti-inflammatory cytokines, including IL-10, tumor growth factor-β (TGF-β), and IL-35. CTLs are CD8^+^ T_EFF_ cells that participate in cellular immunity, and their role is to directly kill infected or malignant cells through the release of cytotoxic cytokines, including TNF-α and IFN-γ, as well as granules including perforin and granzymes^4^.

### Metabolic shifts in T cells

Naive T (T_N_) cells are quiescent and require energy only for survival and circulation. These cells rely predominantly on mitochondrial oxidative phosphorylation (OxPhos) metabolism to generate ATP from glucose or fatty acids (FAs)^11^. Activation of T cells occurs after stimulation of the T cell antigen receptor (TCR) by a ligand in association with costimulatory signals, which initiates several signaling pathways that promote cell differentiation and growth. This activation is associated with a transition from relatively quiescent oxidative metabolism to intense glycolytic metabolism to support energetic needs for proliferation and cytokine production^11–13^. In addition to increased glucose catabolism in activated T cells, glutamine uptake and glutaminolysis are also upregulated to supply the tricarboxylic acid (TCA) cycle for ATP generation^14,15^. The metabolic profile of each specialized T cell subset is optimized to support their unique functions. T_EFF_ cells, including Th1, Th2, and Th17 cells and CTLs, principally rely on aerobic glycolysis and glutaminolysis to promote their rapid growth, proliferation, and effector functions^16,17^. In contrast, Treg cells rely primarily on FA oxidation (FAO) and glutaminolysis to support their suppressive activity^18,19^. Following the primary immune response, a portion of CD4^+^ and CD8^+^ T cells become memory T (T_M_) cells that remain ready to rapidly respond to the same antigen. These cells are long-lived, and until reactivation, they exhibit relatively quiescent oxidative metabolism fueled by FAO, similar to that of T_N_ cells (Fig. 1)^20,21^. Due to competition for glucose and glutamine with cancer cells, tumor-infiltrating T lymphocytes (TILs) are metabolically compromised and functionally exhausted within the tumor microenvironment (TME)^22^. To partially rescue their effector functions, TILs have been shown to increase FAO metabolism as a way to utilize alternative fuel sources^23^.

Increasing evidence indicates that immune cell identity, functions, and metabolism are mediated by overlapping signaling pathways that are controlled, at least in part, via epigenetic and posttranslational modification (PTM) mechanisms. Sirtuins are key epigenetic and PTM regulators in T cells. In this review, we will provide relevant insights into the current understanding of T cell-specific immune response regulation by sirtuins and the therapeutic potential of sirtuin modulators in immune-related diseases.

## Epigenetic mechanisms

In multicellular organisms, all cells contain the same genome; however, gene expression profiles vary from cell to cell, which contribute to differentiation into various cell types within the same organism^24^. This cell type differentiation is mostly regulated by epigenetic mechanisms, which result in dynamic changes in gene expression patterns without changing the DNA sequence^25^. Epigenetic regulation of gene expression is known to occur at the DNA, histone, and RNA levels. In this context, DNA methylation, histone methylation, acetylation, ubiquitination, phosphorylation, and microRNA-dependent gene silencing have been well characterized^26^. Methylation of DNA is mediated by DNA methyltransferases and consists of the addition of a methyl group to cysteine residues within DNA regions that are rich in cysteine-guanine dinucleotides. DNA methylation can act either to inhibit gene transcription if methylation occurs within a promoter region or to promote gene transcription if methylation occurs within the gene body^27^. Noncoding microRNAs are single-stranded RNA fragments that interact with target messenger (m)RNAs via a perfectly complementary base sequence, resulting in mRNA cleavage and translational repression^28^. Another important and common epigenetic mechanism that contributes to gene expression regulation is the modification of histones. Histone acetyltransferases (HATs) and histone deacetylases (HDACs) are the two classes of enzymes involved in the dynamic regulation of histone acetylation^29^. Acetylated histones maintain an open and fluid chromatin structure, called euchromatin, promoting gene transcription, while deacetylated histones often form a tightly packed chromatin structure that prevents gene transcription, called heterochromatin^30^.

## Posttranslational modification

PTM is a biochemical mechanism in which amino acid residues from a protein are covalently modified after translation to regulate protein folding, degradation, signaling, localization, stability, enzymatic activity or protein–protein interactions^31^. There are over 400 known PTM types, including phosphorylation, glycosylation, ubiquitination, nitrosylation, methylation, acetylation, lipidation and proteolysis, which influence almost all aspects of physiological and pathological cell processes^32^. In contrast to epigenetic mechanisms, PTM allows rapid changes in protein properties in response to cellular needs during acute stress phases. In addition, multisite PTM leads to a vast variety of potential molecular states^33^. Phosphorylation is the most widely studied PTM and can regulate signaling pathways, the cell cycle, metabolism, the immune response, and cellular growth and differentiation^34^. Ubiquitin (Ub) is a small and highly conserved protein made of 76 amino acids that can be attached to a lysine substrate through a complex conjugation process requiring a Ub-activating enzyme (E1), Ub-conjugating enzyme (E2) and Ub ligase (E3). Ubiquitination plays important regulatory roles in the life cycle of proteins by leading to the degradation of target substrates^35^. This process can be reversed by deubiquitinating enzymes (DUBs), which remove conjugated Ub molecules from target substrates^36^. Protein acetylation has emerged as a key PTM participating in cellular regulation, particularly through the modification of histones, nuclear transcription regulators and metabolic enzymes^37^. Lysine acetylation is the prevalent modification of metabolic enzymes, and virtually every enzyme in glycolysis, gluconeogenesis, the TCA cycle, the urea cycle, fatty acid metabolism, and glycogen metabolism has been found to be acetylated in human liver tissue^38^. Protein acetylation status is regulated by a highly dynamic equilibrium between HATs and HDACs^39^.

## Overview on sirtuins

The sirtuin proteins are classified within class III HDACs, which require nicotinamide adenine dinucleotide (NAD^+^) as a cofactor for their deacetylase activity. Although initially identified as HDACs, later studies revealed that sirtuins can deacetylate a variety of nonhistone proteins^40^. Moreover, sirtuins show additional enzymatic functions other than deacetylation.

**Fig. 2:**
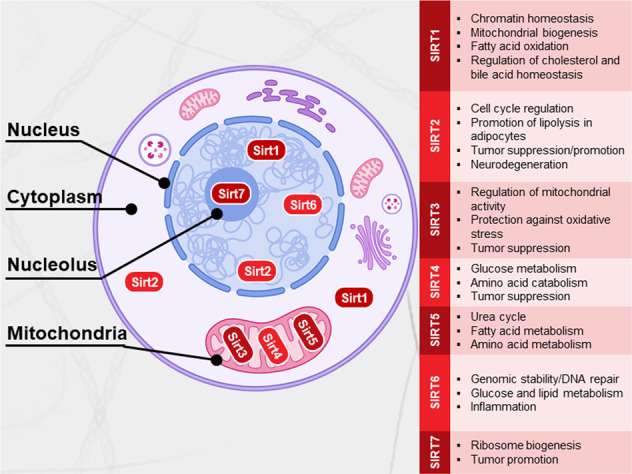
The subcellular localization and main functions of the mammalian sirtuins. Sirt1 is predominantly located in the nucleus and can also be found in the cytosol. Sirt2 is predominantly localized in the cytosol but can shuttle to the nucleus. Sirt3, Sirt4, and Sirt5 are mitochondrial proteins. Sirt6 and Sirt7 are localized in the nucleus and nucleolus, respectively. The main cellular functions are indicated in the boxes.

In mammals, seven sirtuins (Sirt1–7) are ubiquitously expressed with distinct subcellular localization and functions (Fig. 2)^41–43^. Sirt1 is the most studied mammalian sirtuin and is primarily localized in the nucleus. However, under specific conditions, Sirt1 can be transported to the cytoplasm^44^. Sirt2 is a predominantly cytosolic sirtuin that can migrate to the nucleus during mitosis^45^. Sirt6 is exclusively localized in the nucleus, whereas Sirt7 is localized in the nucleolus^41^. All the remaining sirtuins (3–5) are localized in mitochondria^46^. All sirtuins except Sirt5 have mono-ADP-ribosyl transferase activity, whereas Sirt5 catalyzes the removal of succinyl, malonyl, and glutaryl groups from protein targets^47,48^.

The first sirtuin protein was discovered in yeast as silent information regulator 2 (Sir2), whose homolog in mammals is known as Sirt1^49^. Since it was discovered that Sir2 extends yeast lifespan in response to calorie restriction, sirtuins have received considerable attention as anti-aging regulators^50^. Furthermore, sirtuins have been described to regulate a variety of cell processes, including genome stability and cell metabolism^42,43^, and are currently gaining extensive interest for their role in mediating several diseases associated with inflammation^51^, metabolic disorders^52^, and cancers^53^ (Fig. 2).

As sirtuins depend on NAD^+^ (a critical cofactor of metabolism) for their enzymatic activity, any fluctuations in NAD^+^ levels related to nutrient availability affect their functions^54^. Hence, sirtuins serve as energy sensors that directly link environmental signals to cellular metabolic homeostasis. Given the link between metabolic reprogramming, nutrient availability and T cell effector function^55^, sirtuins are speculated to be potential regulators of the immune response, impacting the ability of the immune system to combat foreign pathogens or malignant cells. While each of the sirtuins has been broadly studied and demonstrated to be involved in a number of diseases associated with inflammation^56^, investigations of the roles of sirtuins in adaptive immunity have been limited to certain aspects of the immune response, and the most intense attention has been given to Sirt1 and Sirt3^57^. This review will discuss the latest advances in the roles of the seven mammalian sirtuins in the T cell adaptive response and include our considerations for targeting sirtuins to manipulate the immune response.

## Sirt1

Most studies on Sirt1 have indicated that decreased Sirt1 expression or activity contributes to the enhancement of T cell activation, thereby leading to the occurrence of autoimmune diseases^58^. The immunoregulatory function of Sirt1 is dependent on the cell types and specific substrates that are targeted within the immune response. In addition, Sirt1-mediated regulation of metabolic processes is critical for optimal immune cell function^59^.

c-Jun is upregulated after T cell activation to induce IL-2 production, cell proliferation and differentiation^60^. An early study indicated that Sirt1 inhibits the immune response by blocking c-Jun transcription factor activity, which supports IL-2 production^61^ and thereby decreases Th1 cell activation. Indeed, enhanced expression and activity of Sirt1 induced by resveratrol treatment impeded CD4^+^ T cell activation and IFN-γ production, further confirming that Sirt1 negatively impacts Th1 differentiation and IFN-γ secretion^62^. More recently, in a mouse model of ovarian cancer, Th1 cell differentiation of CD4^+^ T cells was found to be increased after treatment with artesunate, a promoter of microRNA-142 expression that downregulates Sirt1 expression, again confirming the suppressive role of Sirt1 in Th1 cell differentiation^63^.

Studies in Sirt1 knockout (*Sirt1*^−/−^) mice showed that *Sirt1*^−/−^ T cells display increased proliferation and IL-2 production, and *Sirt1*^−/−^ mice are more susceptible to developing autoimmune diseases^64^. Further studies indicated that IL-2 can reverse T cell anergy by suppressing Sirt1 transcription via cytosolic sequestration of its upstream transcription factor, FoxO3a. The expression of a constitutively active form of FoxO3a blocks IL-2-mediated reversal of T cell tolerance by retaining Sirt1 expression^65^.

B-cell lymphoma 2-associated factor 1 (Bclaf1), primarily considered a promoter of cellular apoptosis, has been found to be critical for T cell activation^66^. Kong et al. reported that Sirt1 suppresses Bclaf1 activity by deacetylating histone lysine residues at the Bclaf1 promoter region, resulting in decreased IL-2 gene transcription. Accordingly, *Sirt1*^−/−^ T cells displayed higher expression of the Bclaf1 gene and IL-2 production, and specific knockdown of Bclaf1 reversed the increase in IL-2 production and proliferation observed in *Sirt1*^−/−^ T cells^67^.

Early studies using ovalbumin-induced asthma models in mice demonstrated that pharmacologic inhibition of Sirt1 reduced allergic reactions compared with mock treatment^68,69^. Furthermore, Sirt1 inhibition was found to suppress the differentiation of Th2 cells through the B cell lymphoma/leukemia 11B (Bcl11b) transcriptional activator. Bcl11b is essential for Th2 differentiation, and mice lacking Bcl11b in mature T cells have a diminished capacity to mount Th2 responses during helminth infection and allergic asthma^70^. Sirt1 interacts directly with Bcl11b and is recruited to the promoter template in a Bcl11b-dependent manner to deacetylate histones, leading to transcriptional repression^71^.

Hypoxia-inducible factor 1-alpha (HIF-1α) activity has consistently been associated with the generation of proinflammatory cytokines and restriction of anti-inflammatory cytokines^72^. Sirt1 inhibition was found to promote Th9 cell differentiation and IL-9 production via the Sirt1-mTOR-HIF-1α axis^73^. In the same study, Wang *et al*. indicated that Sirt1-dependent regulation of glycolysis was critical for directing the differentiation of Th9 cells, highlighting the importance of metabolic reprogramming in controlling T cell fate.

The effects of Sirt1 on Th17 cells are controversial, and more studies are needed to dissect its precise role. A few studies have indicated that the differentiation of Th17 cells is affected by the degree of STAT3 deacetylation. Sirt1 activators such as metformin have been shown to impede Th17 cell differentiation and reduce IL-17A and RORγt expression via deacetylation of the STAT3 transcription factor. STAT3 deacetylation restricts its ability to translocate into the nucleus to induce RORγt transcription^74^. Another study showed that in vivo activation of Sirt1 using NAD^+^ supplementation delayed the onset of experimental autoimmune encephalomyelitis (EAE). This protection was hypothesized to be a result of the decrease in Th17-mediated inflammatory responses induced by enhanced Sirt1 expression^75^. Moreover, treatment with methylene blue, another Sirt1 activator, alleviated Th17 responses and significantly reduced the clinical scores of EAE in mice^76^. In contrast, other researchers have shown that Sirt1 activation promotes the Th17 phenotype via RORγt deacetylation. Deacetylated RORγt appears to have stronger transcriptional activity than Foxp3, thus strengthening the Th17 proinflammatory phenotype and suggesting that Sirt1 inhibitors may protect against autoimmune diseases^77^.

Foxp3 is a master regulator of Treg cell development and function and has three lysine acetylation sites (K31, K262, and K267) targeted by Sirt1^78^. Hyperacetylation of Foxp3 prevents its polyubiquitination and proteasomal degradation. Accordingly, Sirt1 deacetylase activity has been found to reduce Foxp3 protein levels, and treatment with Sirt1 inhibitors results in increased functional Treg cells^79^. In addition to the posttranslational control of Foxp3 by Sirt1, some studies reported that genetic deletion or pharmacologic inhibition of Sirt1 increased the number and suppressive activity of Foxp3^+^ Treg cells by increasing Foxp3 mRNA levels^78,79^. In contrast, Sirt1 was reported to promote Treg cell survival by stabilizing the Notch1 intracellular domain proximal to the membrane, given that the Notch receptor is essential for Treg cell survival within caloric-restricted conditions^80^.

Basic leucine-zipper ATF-like transcription factor (BATF) regulates multiple aspects of the T cell immune response^81^. Sirt1 was reported to impact CD8^+^ T cell differentiation and effector functions under the influence of BATF. Indeed, BATF has been shown to transcriptionally inhibit Sirt1 expression, resulting in increased histone acetylation at the T-bet locus. In turn, high levels of T-bet expression promote CD8^+^ T cell differentiation, and loss of BATF consequently inhibits CD8^+^ T cell differentiation^82^.

**Fig. 3:**
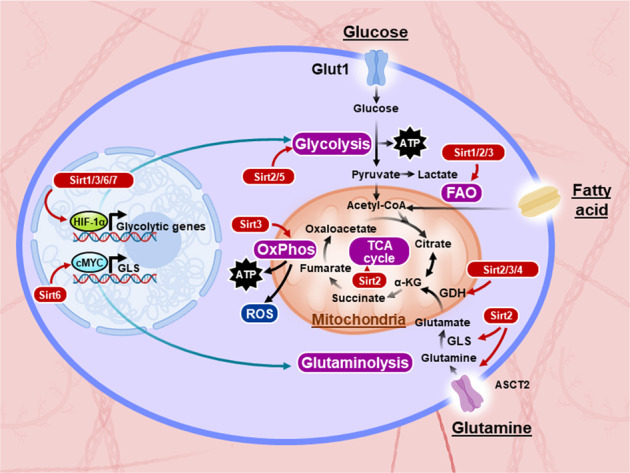
Mammalian sirtuins and metabolic reprogramming. The entry of glucose into the cell is mediated by the glucose transporter Glut1. Glucose is then metabolized to pyruvate, which enters the mitochondrial tricarboxylic acid (TCA) cycle and generates reducing equivalents for ATP production via oxidative phosphorylation (OxPhos). During aerobic glycolysis, pyruvate is fermented into lactate in the cytoplasm despite the availability of oxygen for complete glucose oxidation; this process is called the Warburg effect. Glutamine enters cells using alanine, serine, cysteine-preferring transporter 2 (ASCT2), and it is converted into glutamate by glutaminase (GLS) and into α-ketoglutarate (α-KG) by glutamate dehydrogenase (GDH), which enters the TCA cycle for ATP production via OxPhos. Fatty acid molecules are catabolized into acetyl-CoA via fatty acid oxidation (FAO), and acetyl-CoA enters the TCA cycle for ATP production. Sirtuins are metabolic sensors that modulate a variety of metabolic pathways. Sirt1, Sirt3, and Sirt6 restrain the glycolytic pathway through hypoxia-inducible factor 1-alpha (HIF-1α) inhibition or direct effects. Sirt3 upregulates the OxPhos pathway by enhancing the activity of mitochondrial complexes I, II, and III and dampening reactive oxygen species (ROS) production. Sirt1 is also able to increase FAO by activating PPAR-α and PGC1, while Sirt3 upregulates FAO under caloric restriction conditions. Sirt2 deacetylates and inhibits the activity of many metabolic enzymes involved in glycolysis, glutaminolysis, the TCA cycle and FAO. Sirt3 and Sirt4 activate and inhibit glutaminolysis, respectively, by regulating GDH activity. Sirt6 impacts the glutaminolysis pathway by regulating c-Myc transcriptional activity. Sirt5 increases glycolysis by increasing the activity of the glycolytic enzyme GAPDH. Sirt7 can also repress HIF-1α and, therefore, may inhibit the transcription of glycolytic genes.

The metabolic switch toward FAO is a characteristic of T_M_ cell differentiation, and this switch is known to be supported by the transcriptional coactivator PGC-1, which is involved in mitochondrial biogenesis and OxPhos metabolism^83^. Sirt1-mediated deacetylation has been shown to increase the transcriptional activity of the PGC-1α and PGC-1β cofactors^84,85^, suggesting a potential role for Sirt1 in promoting T_M_ cell formation. A previous report indicated a decrease in Sirt1 expression levels in terminally differentiated CD8^+^CD28^−^ T_M_ cells, which concomitantly displayed enhanced glycolytic and cytotoxic capabilities^86^, suggesting that Sirt1 may restrain glycolytic metabolism in T cells (Fig. 3).

## Sirt2

Until recently, our knowledge on the role of Sirt2 within the immune system was limited to its anti-inflammatory function via negative regulation of the NF-κB p65 subunit^87^. Accordingly, Sirt2-deficient mice were found to develop severe forms of dextran sodium sulfate (DSS)-induced colitis via polarization of bone marrow-derived macrophages^88^. Furthermore, that study observed a greater proportion of activated CD4^+^CD69^+^ T cells in the mesenteric lymph nodes of *Sirt2*^−/−^ mice in response to DSS-induced colitis, suggesting a role for Sirt2 in limiting CD4^+^ T_EFF_ cell functions^88^.

A recent publication uncovered a new function of Sirt2 as a master regulator of T cell metabolism and functions. Hamaidi et al*.* found increased proliferation of CD4^+^ and CD8^+^ *Sirt2*^−/−^ T cells upon TCR activation and activated CD8^+^ T cells displayed increased IFN-γ production and granzyme B expression, leading to superior cytotoxic activity. This hyperreactive phenotype of *Sirt2*^−/−^ T cells endowed mice with superior antitumor immunity upon tumor challenge in vivo^89^.

Mechanistically, this hyperactivation and enhanced function of *Sirt2*^−/−^ T cells is linked to Sirt2 regulation of multiple metabolic pathways that play crucial roles in T cell effector functions. Sirt2 deacetylates and negatively impacts the activity of key metabolic enzymes of the glycolysis, TCA cycle, FAO and glutaminolysis pathways. Accordingly, Sirt2 deletion during T cell activation was followed by hyperacetylation of multiple metabolic enzymes and amplification of their activities, leading to increased aerobic glycolysis, OxPhos, FAO, and glutaminolysis (Fig. 3)^89^.

Sirt2 was found upregulated in T_M_ cell stages. T_M_ cells rely on FAO metabolism, and Sirt2 negatively impacts FA catabolism; thus, Sirt2 deletion promoted CD4^+^ and CD8^+^ T_M_ cell formation ex vivo, which was associated with superior cell survival and decreased apoptosis. Consistently, increased accumulation of T_M_ cells within the TME and the secondary lymphoid organs of *Sirt2*^−/−^ mice was observed upon tumor challenge, whereas no qualitative or quantitative phenotypic differences were observed between unchallenged WT and *Sirt2*^−/−^ control mice^89^.

That study investigated the role of Sirt2 during T cell activation and maturation and within the antitumor immune response^89^. However, the role of Sirt2 in different CD4^+^ T cell subsets with distinct metabolic programs, including Treg, Th1, Th2, Th9 and Th17 cell subsets, requires further investigation.

## Sirt3

Sirt3 localizes mainly to the mitochondrial matrix and plays an important role in regulating elements of mitochondrial metabolism, including the TCA cycle, urea cycle, FAO, and reactive oxygen species (ROS) detoxification (Fig. 3)^90^. Sirt3 promotes energy generation; thus, its expression is higher in metabolically active tissues.

A recent report indicated that T cells from Sirt3-deficient (*Sirt3*^−/−^) donor mice caused reduced graft-versus-host disease (GVHD) severity in comparison to T cells from control donor mice, suggesting that Sirt3 targeting can improve transplant recipient outcome. In that study, the protective effect of allogeneic *Sirt3*^−/−^ T cells was related to reduced T cell proliferation and CXCR3 expression, with no significant impact on cytokine secretion or cytotoxic functions^91^.

Furthermore, Sirt3 deficiency in mouse models had no impact on immune responses against bacterial and fungal infections^92^, suggesting that Sirt3 may play a limited role in T_EFF_ cell functions.

Inhibition of OxPhos impairs Treg cell function, and given the key role of Sirt3 in OxPhos, it is expected that Sirt3 promotes the suppressive activity of Treg cells. In fact, Treg cells from *Sirt3*^−/−^ mice exhibited impaired suppressive functions, as demonstrated in in vitro suppression assay and an in vivo allograft model, and HDAC9 deletion was found to increase Treg suppressive activity by increasing Sirt3 expression^93^.

Sirt3 is also involved in CD8^+^ T cell function. *Sirt3*^−/−^ CD8^+^ T cells exhibited reduced ROS production and CXCR3 expression upon activation. Moreover, T cells from *Sirt3*^−/−^ donor mice were able to reduce GVHD within the gastrointestinal tract, which is probably due to decreased CXCR3-dependent CD8^+^ T cell trafficking to the site^93^.

## Sirt4

Sirt4 is a mitochondrial sirtuin with ADP-ribosylation activity. No deacetylase activity of Sirt4 has yet been identified. By ADP-ribosylating glutamate dehydrogenase (GDH), Sirt4 represses its enzymatic activity, which limits the metabolism of glutamine into glutamate to generate ATP (Fig. 3)^94^. It is thus conceivable that T cell metabolic activity and effector functions can be enhanced by Sirt4 inhibition via increasing glutaminolysis, another ATP-generating pathway used by activated T cells. Furthermore, given the role of glutaminolysis in Th17 cell differentiation^95^ and Treg cell development^18,19^, we would also expect a proinflammatory phenotype with Sirt4 deficiency. Sirt4 was found to physiologically break immune tolerance and to resolve acute inflammation in a model of sepsis by coordinately reprogramming the metabolism and bioenergetics of human monocytes^96^.

Recently, in a mouse model of neuroinflammation following traumatic spinal cord injury, Sirt4 expression was found to be upregulated in infiltrating Treg cells in the spinal cord parenchyma^97^. Interestingly, Sirt4 overexpression in splenic naive Treg cells was found to alleviate the expression of Foxp3, IL-10, and TGF-β and to weaken the inhibitory activity of Treg cells. Additionally, Sirt4 overexpression blocked in vitro inducible Treg cell generation from conventional T cells^97^. Consistently, Sirt4 knockdown increased the anti-inflammatory activity of infiltrating Treg cells in the parenchyma of injured spinal cords^97^. The authors concluded that Sirt4 inhibits the anti-neuroinflammatory activity of Treg cells by blocking AMPK signaling given that an AMPK agonist restored the expression of Foxp3 and IL-10 in Treg cells^97^. However, this result could also be related to Sirt4’s impact on glutamine metabolism and consequently on Treg cell development.

## Sirt5

Sirt5 is another mitochondrial sirtuin that displays a weak deacetylase activity. However, Sirt5 is unique in executing novel enzymatic activities involving lysine desuccinylation, demalonylation, and deglutarylation^98^. Given the emerging role of protein succinylation in the immune response^99^, future studies focusing on the role of Sirt5 in T cell function are needed.

An anti-inflammatory protective role of Sirt5 was reported in a DSS-induced colitis mouse model. Sirt5 was found to suppress IL-1β production and proinflammatory responses in macrophages by regulating pyruvate kinase M2 (PKM2) succinylation. Sirt5-dependent succinylation promotes PKM2 entry into the nucleus, where the PKM2-HIF1α complex is formed at the promoter of the IL-1β gene to stimulate its transcription^100^. Paradoxically, proinflammatory activity of Sirt5 was described during the acute and immunosuppressive phases of sepsis. The induction and persistence of a hypoinflammatory and immunosuppressive state in severe sepsis are commonly associated with increased risks of secondary infections and mortality. Sirt5 was found to rescue the innate inflammatory response of endotoxin-tolerant macrophages by promoting acetylation of NF-κB p65. Mechanistically, Sirt5 competes with Sirt2 to interact with NF-κB p65 and block its deacetylation by Sirt2, which consequently leads to increased acetylation of p65 and activation of the NF-κB pathway with its downstream cytokines^101^.

Although little work has been done to study Sirt5 in the adaptive immune response, a recent study has demonstrated a pivotal role of Sirt5 in regulating T cell activation and differentiation^102^. Indeed, Sirt5 deficiency was found to promote mouse naive T cell activation and increased IFN-γ production upon TCR ligation and to further influence T cell differentiation. Sirt5 deletion enhanced Th1 cell and CTL differentiation and decreased CD4^+^ Treg cell differentiation, whereas no qualitative or quantitative phenotypic differences were observed in the peripheral lymphoid organs of WT and *Sirt5*^−/−^ mice under steady state conditions^102^. More importantly, even though *Sirt5*^−/−^ mice were found to be highly susceptible to DSS-induced colitis^100^, *Sirt5*^−/−^ mice were resistant to colorectal tumorigenesis following experimentally induced colitis, and this resistance was related to increased IFN-γ production in the colon tissue by immune cells^102^. While these data indicate the importance of Sirt5 in T cell activation and antitumor functions, the molecular mechanisms of Sirt5 activity need to be further elucidated.

Remarkably, studies on a large panel of preclinical mouse models of sepsis showed that Sirt5 deficiency has no impact on antimicrobial host immune defenses^103^, suggesting that Sirt5 plays a limited role in T_EFF_ cell functions. On the other hand, these observations support the assumption that therapies directed against Sirt5 do not impair antibacterial host defenses.

## Sirt6

Sirt6 is a chromatin-associated sirtuin implicated in numerous biological functions, including transcriptional repression, glucose homeostasis, DNA repair, telomeric function, cellular differentiation, mitosis, and meiosis^104^. Although Sirt6 has mono-ADP-ribosylase activity, the most robust activity of Sirt6 is histone deacetylation. Sirt6 deacetylates histone H3 lysine 9 (H3K9), H3K18, and H3K56 to induce transcriptional repression. Sirt6 can also remove the long-chain fatty acyl group from lysine^105^.

Recent studies have revealed that Sirt6 possesses anti-inflammatory properties. Sirt6 represses proinflammatory gene transcription by affecting chromatin structure rather than by directly deacetylating NF-κB, while Sirt1 and Sirt2 directly deacetylate p65 and inhibit its transcriptional activity^87,106^. Although Sirt6 physically associates with the p65 subunit, it modulates its transcriptional activity by deacetylating H3K9 on the promoter of selected NF-κB target genes^107^. Sirt6 was also reported to interact with JUN and deacetylate H3K9 at the promoter of proinflammatory genes whose expression involves JUN. The same study also indicated that Sirt6-null mice develop chronic liver inflammation attributable to Sirt6 deficiency in immune cells and macrophages, and these cells express increased levels of MCP1, IL-6, and TNF^108^. While Sirt6 appears to exert anti-inflammatory action when acting at the transcriptional level, scattered evidence seems to suggest that Sirt6 induces proinflammatory effects by modulating distinct intracellular signaling factors, such as Ca2^+^ homeostasis^109^ and mRNA translation^110^. Indeed, Sirt6 has been shown to promote the expression of cytokines and chemokines, including IL-6, TNF, CXCL2, and IFN-γ, and to positively regulate TNF secretion by increasing mRNA translational efficiency in several immune cells^110–112^.

Little is known about the role of Sirt6 in the adaptive immune response. HIF-1α is a transcriptional regulator of glycolysis and is known to regulate the production of several cytokines^113^ and to enhance Th17 cell differentiation while concomitantly inhibiting Treg cell function^114,115^. Furthermore, Myc is a global regulator of the immune response and plays a crucial role in glutamine metabolism^15^. Glycolysis and glutaminolysis are critical metabolic pathways on which T_EFF_ cells depend to meet their energy needs. Dynamic switching between these metabolic pathways needs to be tightly regulated to achieve optimal function within immune cells.

Sirt6 appears to strongly oppose glycolysis by inhibiting the transcription of many glycolytic genes^116^. Sirt6 has been shown to physically interact with HIF-1α and to corepress its transcriptional activity by deacetylating histone H3K9 at the promoters of several glycolytic genes^117^. Furthermore, Sirt6 also regulates glutaminase expression, thus modulating the glutaminolysis pathway. Sirt6 interacts with Myc and reduces its transcriptional activity by deacetylating histones at the promoter region of Myc target genes^118^. Collectively, these observations suggest that Sirt6 potentially has an immunomodulatory effect on T cell metabolism and functions by regulating the HIF-1α and Myc pathways (Fig. 3).

## Sirt7

Sirt7 is the last and least studied member of sirtuins. Sirt7 is a vital regulator of ribosome biogenesis, and it is enriched in nucleoli, where it facilitates RNA polymerase I-dependent transcription of ribosomal (r)RNA genes^119^. Sirt7 expression is linked to cell proliferation and oncogenic activity, connecting Sirt7-dependent regulation of ribosome biogenesis with cell cycle progression, metabolic homeostasis, stress resistance, aging and tumorigenesis^120–122^.

Recent studies suggest that Sirt7 is also involved in inflammation. Vakhrusheva et al. showed that Sirt7 deletion predisposes mice to heart hypertrophy and cardiac inflammation. These authors observed an increased infiltration of immune cells, with higher levels of proinflammatory cytokine production^123^. Information regarding the possible involvement of Sirt7 in the adaptive immune response is currently very limited.

## Concluding remarks and future perspectives

The well-documented capacity of sirtuins to promote the resolution of the inflammatory response has led many research groups to focus on developing therapeutic strategies to target their enzymatic activity in vivo. However, to achieve the desired clinical outcomes, it is paramount to decide whether to stimulate or block sirtuin activity and how to modulate a specific sirtuin within a specific T cell subset. All these challenges are worthy of further study due to the ubiquity of sirtuins. In addition, these proteins have intricate and partially unknown functions within an organism, reflecting the immense challenge of targeting any of them.

For example, enhancing Treg cell suppressive activity with Sirt3 agonists to attenuate inflammation represents a potential strategy to treat autoimmunity. Similarly, the use of Sirt1 activators to suppress inflammation could help treat autoimmune diseases. Metformin, classically prescribed for the management of type 2 diabetes, appears to have a stimulatory effect on sirtuins. Sirt1 activation by metformin has been shown to decrease inflammation by decreasing Th17 cell differentiation^74^. Resveratrol, a compound with sirtuin-activating effects, is currently of intense interest within both scientific and lay communities for its anti-inflammatory properties^124^. Resveratrol stimulates Sirt1 activity, which decreases c-Jun acetylation, consequently restraining T cell activation^62^. Resveratrol has been shown to improve outcomes in several experimental autoimmunity models^125^. For instance, administration of resveratrol protected mice against experimental rheumatoid arthritis by inhibiting Th17 cell differentiation^126^. Furthermore, resveratrol was proven to have both preventative and therapeutic effects by reverting the advanced stages of insulitis in a nonobese diabetic (NOD) mouse model due to reduced Th17 cells^127^. Moreover, resveratrol induced protective effects against high-fat diet-induced obesity in mice in part by increasing total Treg cells^128^.

Despite the obvious progress in the organ transplantation field, the commonly required immunosuppressive therapies are known to ultimately induce cancer and quite often infections. Presently, sirtuins are being thoroughly studied for their role in promoting organ transplant tolerance. Treg and Th17 cells drive transplant tolerance, and in relation to these T cell subsets, Sirt1 has emerged as an eligible target for clinical interventions in transplantation. Indeed, Sirt1 deletion improved Treg cell suppressive activity in vitro and in vivo, and mice with specific Sirt1 deletion in Foxp3^+^ Treg cells exhibited prolonged survival after cardiac allografts concomitant with increased Treg cell infiltration. In the same study, similar results were obtained when using Sirt1 inhibitors in vivo^129^. Later, comparable findings were reported after kidney transplantation from BALB/c mice into C57BL/6 recipient mice treated with EX-527, a Sirt1 inhibitor^130^.

Recently, the effect of sirtinol, a Sirt1 inhibitor, on heart allograft survival was evaluated, and it was found that sirtinol-treated recipients exhibited increased numbers of Foxp3 Treg cells and reduced numbers of Th17 cells in addition to decreased expression of the IL-17A and RORγt transcription factor^131^. Therefore, Sirt1 inhibition might affect the Th17/Treg cell ratio in favor of Treg cells. In contrast, Sirt1 activators such as metformin were found to decrease IL-17A and RORγt expression in mouse models as described earlier^74^. Certainly, more studies are required to define the appropriate action of Sirt1 targeting for allograft retention.

To date, the research community has focused on promoting the anti-inflammatory activity of sirtuins to treat a multitude of autoimmune diseases and other chronic diseases associated with inflammation. However, we must extend our interest toward blocking the activity of sirtuins to promote the effector activity of T cells in the context of antitumor immunity. Indeed, enhancing tumor rejection by boosting tumor-reactive T cell activity would be tremendously effective in fighting cancer. Recent reports demonstrating the immunotherapeutic potential of Sirt2 and Sirt5 blockade in T cells to reject tumor challenge^89^ and to protect against colorectal tumorigenesis in murine models, highlight the expected effectiveness of such strategy^102^. In addition, sirtuins have been shown to play an oncogenic role in many tumor types^132^, and sirtuin inhibitor drugs have been shown to have tumor-suppressive activity on cancer cells, suggesting an intriguing possibility that in certain cancer patients, targeting sirtuins may suppress tumor proliferation while simultaneously boosting antitumor immunity. Alternatively, manipulation of sirtuins could be achieved by gene editing during TIL expansion or chimeric antigen receptor (CAR) T cell generation ex vivo, thus selectively targeting T cells in the context of immune cell therapy^133,134^.

Many reports indicate that exhausted tumor-infiltrating lymphocytes display dysregulated metabolism within the metabolically restricted tumor microenvironment^22,23,135,136^. Modulation of sirtuins to enhance the metabolic activity of T cells could provide a viable strategy to restore exhausted tumor-reactive T cells.
